# Performance of Multidrug-Resistant Tuberculosis Surveillance in Yemen: Interview Study

**DOI:** 10.2196/14294

**Published:** 2019-10-03

**Authors:** Jihan Abdulmughni, Esam Mohammed Mahyoub, Abdulaziz Thabit Alaghbari, Abdulwahed Abdelgabar Al Serouri, Yousef Khader

**Affiliations:** 1 Yemen Field Epidemiology Training Program Sana'a Yemen; 2 National Tuberculosis Control Program Sana'a Yemen; 3 Jordan University of Science and Technology Amman Jordan

**Keywords:** multidrug-resistant tuberculosis, surveillance evaluation, Yemen, field epidemiology program

## Abstract

**Background:**

Multidrug-resistant tuberculosis (MDR-TB) is a major challenge to ending TB occurrence by 2035. In Yemen, the 2011 survey showed an MDR-TB prevalence of 1.4% among new cases and 14.4% among previously treated cases. The National Tuberculosis Control Program (NTCP) established four MDR-TB sentinel surveillance sites in 2013 to monitor the MDR-TB situation. In Yemen, the 2011 survey showed an MDR-TB prevalence of 1.4% among new cases and 14.4% among previously treated cases. The NTCP established four MDR-TB sentinel surveillance sites in 2013 to monitor the MDR-TB situation.

**Objective:**

This study aimed to assess the performance of MDR-TB surveillance and determine its strengths and weaknesses.

**Methods:**

We used the updated Center for Diseases Control and Prevention guidelines for evaluating public health surveillance systems. Interviews were conducted with NTCP managers and Regional MDR-TB centers’ staff using a semistructured questionnaire. We used a 5-point Likert scale to assess the usefulness and other attributes (eg, simplicity and flexibility). The mean percentage was calculated for each attribute and used for the final rank of the performance: poor (<60%), average (60%-80%), and good (>80%).

**Results:**

The MDR-TB surveillance system achieved good performance in usefulness (87%), acceptability (82%), and data quality (91%); average performance in flexibility (61%) and simplicity (72%); and poor performance in stability (55%). The overall performance score was average (74%). Although strong commitment, good monitoring, and well-trained staff are the main strengths, depending on an external fund is a major weakness along with unavailability of the MDR-TB unit at the governorate level.

**Conclusions:**

Although the MDR-TB surveillance system has achieved an average overall performance, more efforts are required to improve its stability by ensuring constant power supply to enable laboratories to perform necessary diagnostic and follow-up tests. Gradual replacement of donors’ funds by the government is recommended. Scaling up of MDR-TB services and removing access barriers are crucial.

## Introduction

Tuberculosis (TB) is one of the major causes of morbidity and mortality worldwide, with more than 10 million newly reported cases and 1.7 million deaths in 2015 [1]. With an estimated 480,000 new multidrug-resistant (MDR) TB cases every year, developing drug resistance to anti-TB drugs becomes a major challenge for the global prospect of ending TB by 2035 [2]. Although MDR-TB is defined as the resistance to rifampicin and isoniazid, extensively drug-resistant TB (XDR-TB) is an MDR-TB with resistance to fluoroquinolone and at least one of the injectable second-line anti-TB drugs [3]. As MDR-TB is more difficult and costly to treat, there are increasing concerns about its continued spread and negative impact on the population and health systems [4]. Effective response to MDR-TB should not only focus on treating drug-susceptible tuberculosis, but also include strong surveillance systems, drug susceptibility testing for all patients with tuberculosis, rapid linkage to effective treatment, and patient-centered care throughout the treatment course [5].

Globally, in 2015, 4% of all new patients and 20% of previously treated patients with TB had MDR-TB. China, India, and the Russian Federation account for nearly half of the global MDR-TB prevalence [6]. The data on MDR‐TB from eight countries of the Eastern Mediterranean Region (Egypt, Islamic Republic of Iran, Jordan, Lebanon, Morocco, Oman, Qatar, and Yemen) showed an MDR-TB prevalence of 2.0% among new cases and 35.3% among previously treated cases [7]. Although most countries in the region had established MDR-TB management in line with World Health Organization (WHO) guidance, it estimated that the region has only detected 12% of MDR-TB cases and has enrolled 72% of them on treatment.

In Yemen, TB is the fourth biggest public health problem, with an incidence of 48 per 100,000 people [8]. However, there were no systems in place for the management of MDR in Yemen before 2013, as shown by two surveys performed in 2005 and 2011. The 2004-2005 survey showed an MDR-TB prevalence of 2.9% among new smear-positive cases and 11.3% among previously treated cases compared to 1.4% and 14.4%, respectively, in the 2010-2011 survey [9]. By the end of 2013, the National TB Control Program (NTCP) started the DR-TB Management and Surveillance program. This study aimed to evaluate the performance of MDR-TB surveillance in Yemen and determine its strengths and weaknesses.

## Methods

### Evaluation Design

A descriptive evaluation study was conducted to assess the performance of MDR-TB surveillance system in Yemen using the Updated CDC guideline for evaluation of public health surveillance system [8]. The study was conducted at Sana’a city from November to December 2016. Data on MDR-TB were collected from the NTCP and the four Regional MDR-TB centers.

### The Multidrug-Resistant Tuberculosis Surveillance System

The MDR-TB is a sentinel system through four regional MDR-TB centers at Sana’a City, Aden, Taiz, and Al Hodaidah, with an aim to cover the whole country. The centers provide detection, diagnosis, treatment, and follow-up through a community-based outpatient treatment-delivery strategy that consists of a community supporter, community nurse, drug provider, coordinator, and lab technician. The community supporter is one of the patient’s family members who observes the patient daily, has contact with the community nurse regularly, and fills monthly reports for daily treatment intake. The community nurse follows the community supporters on a monthly basis, directly contacts patients during the follow-up visits, and is responsible for registration of DR-TB. The drug provider is a nurse responsible for supplying drugs to patients with MDR-TB and for the drug supply registry. All patients have monthly clinical assessments by a specialist doctor. The lab technician is responsible for performing the smears and cultures or others lab tests in addition to completing the lab registry and reports. The MDR-TB coordinator is responsible for sending the reports to the NTCP. All centers have equipped labs for drug susceptibility testing (DST) and culture. Due to a total breakdown of the electricity network that is needed for DST, the regional MDR-TB centers were provided with the GenXpert, except for the Taiz MDR-TB center, which still sends samples to Sana’a City for diagnosis.

At the MDR-TB centers, there are three hard-copy registers: the DR-TB case register, the lab register, and the MDR-TB drug supply register. The DR-TB cases register contains a set of variables including demographic variables, previous TB registration number, type of drug resistance, starting category 4 treatment date, and use of previous second-line drugs, DST result, smear and culture follow-up results, and HIV status.

### Data Collection and Analysis

The MDR-TB documents were reviewed to describe the system. The mangers of the NTCP were interviewed to assess usefulness, flexibility, and stability of the MDR-TB system. Semistructured questionnaires were used to collect data from staff of the four MDR-TB centers to evaluate simplicity and acceptability of the system. Specific items were used to evaluate each performance attribute. Respondents from the four centers were requested to rate the items measuring the system usefulness as well as the simplicity, flexibility, acceptability, and stability using the 5-point Likert scale (1=strongly disagree, 2=disagree, 3=not sure, 4=agree, 5=strongly agree). The mean percentage score was calculated for each attribute, where a higher score indicates a higher level of performance. The performance was interpreted using the following scoring system that was used in previous similar studies: <60%, poor; 60%-80%, average; and >80%, good [10-12]. To assess the data quality, we reviewed 10% of randomly selected MDR-TB cases’ files and registers. The selected files were reviewed for any missing data or discrepancies between data on cases’ files and register. Epi info version 7.2 (Centers of Disease Control and Prevention, Atlanta, Georgia) was used for data entry and analysis.

## Results

### Data Flow of Multidrug-Resistant Tuberculosis Surveillance

**Figure 1:**
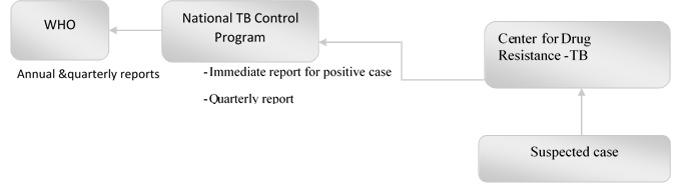
Data flow of multiple drug-resistant tuberculosis cases in Yemen. WHO: World Health Organization; TB: tuberculosis.

Figure 1 shows the data flow of the MDR-TB surveillance system, where the suspected case is sent to the nearest regional DR-TB center for lab confirmation and treatment. According to the tests results, the lab sends immediate reports for positive cases to the NTCP to be included in their drug supply for the reporting regional DR-TB. Each regional DR-TB center sends quarterly reports to the NTCP regarding MDR-TB cases during that quarter including lab report, drug supply, and treatment outcome. The NTCP sends quarterly and annual reports to the WHO.

### Usefulness

The NTCP managers agreed that the data are useful. They reported that they used the data to estimate needs of the NTCP in terms of drugs and lab tests. Furthermore, all managers agreed that the MDR-TB data are used to estimate the TB magnitude, incidence, and mortality rates in order to monitor the trend of MDR-TB over time; identify areas at greater risk; update and develop the strategic direction for MDR-TB activity; and plan the resources for detection, prevention, and control activities. The usefulness percentage score was 87%, which indicates that the system is useful (Table 1).

### Flexibility

All NTCP managers agreed that the system is able to adapt any change in case definition. Four managers (80%) reported that the system is flexible and can be integrated with other surveillance systems. For example, the system is integrated with the HIV program, where all MDR-TB cases are tested for HIV, and positive cases are reported to the HIV program. The flexibility score of the system was 61%, indicating average flexibility (Table 2).

**Table 1:** The usefulness of the multidrug-resistant tuberculosis surveillance system as assessed by the five managers of the National Tuberculosis Control Program in Yemen.

Statements	Score	Proportion	Rank
The MDR-TB^a^ data are used to estimate the MDR-TB magnitude, incidence, and mortality rates	4.4	88	Good
The data are used to monitor the trend of MDR-TB spread over time	4.0	80	Good
The data are used to identify areas at greater risk	4.2	84	Good
The data are used for planning the resources for detection, prevention, and control activities	4.6	92	Good
The data are used to update and develop the strategic direction for MDR-TB activity	4.4	88	Good
The collected data are used to estimate the drugs and laboratory test needed	5.0	100	Good
The data are used to identify research priorities	4.0	80	Good
Average	4.4	87	Good

^a^MDR-TB: multidrug-resistant tuberculosis.

**Table 2:** The flexibility of the multidrug-resistant tuberculosis surveillance system as assessed by the five managers of the National Tuberculosis Control Program in Yemen, 2016.

Statements	Score	Proportion	Rank
The system can be adapted to accommodate addition/change to case definitions.	4.0	80	Good
The system can be integrated with other surveillance such as HIV	3.6	72	Good
The system is not affected by outside funding	1.6	32	Poor
Average	3.1	61	Average

### Stability

All participants said that unscheduled outages have been rarely occurred before the war. Three (60%) managers said that the system would collapse if the donor fund stops. The stability of the system was poor, as evidenced by the stability score of 55% (Table 3).

### Weakness and Strength of the Multidrug-Resistant Tuberculosis Surveillance System

According to the NTCP managers, the strengths of the system include the program’s commitment to provide a treatment for cases, good monitoring system, well-trained staff, and strong donor support. On the other hand, the managers stated that dependence on the external fund is a major weakness of the system, especially if stopped. Other weaknesses included unavailability of the MDR-TB center at the governorate level, absent inpatient department, no refresher training or training for new staff, absence of an electronic system, no trained staff for data analysis at the regional MDR-TB centers levels, and no constant electrical power supply for the lab.

### Simplicity

All the 16 regional MDR-TB staff agreed that the case definitions are easy and clear. Although 71% reported that the guidelines are easy and understandable, 50% reported that it is preferable to have these guidelines written in Arabic. Regarding data collection, 44% respondents reported that this is difficult because, sometimes, the data are needed to call the patient and 66% reported that the data require regular follow-up. All the respondents agreed that transferring data to the NTCP is easy. However, 56% of the respondents reported that there is shortage in lab tests, especially the routine follow-up lab tests (eg, liver function, renal function, and eye and hearing tests) that are only available at the Sana’a regional MDR-TB center. The majority (94%) stated that they received training only once in 2014, and all stated that they need refresher training. The simplicity attribute has been scored 72%, which means that the system is average in simplicity (Table 4).

**Table 3:** The stability of the multidrug-resistant tuberculosis surveillance system as assessed by the five managers of the National Tuberculosis Control Program in Yemen.

Statements	Score	Proportion	Rank
Unscheduled outages/electrical power off rarely occur	1.4	28	Poor
You have your own source	1.6	32	Poor
There is planned resources for maintenance of the system	4.0	80	Poor
The system is stable even with cutting foreign fund	1.4	28	Poor
Trained staff rarely turnover	4.0	80	Good
Data release regularly	4.0	80	Good
Average	2.7	55	Poor

**Table 4:** The simplicity of the multidrug-resistant tuberculosis surveillance system as assessed by 16 service providers in Yemen.

Statements	Mean score	Percentage score	Rank
Case definition is available and easy to apply	4	80	Good
You have easy guide to use	3.5	70	Average
Registers or form is easy to fill	4	80	Good
Collecting case detailed information don’t need telephone or visit	3	60	Poor
Data compiling time, place and person.	4	80	Good
Collecting data do not need much time	3	60	Poor
You received training on MDR^a^ surveillance	3.8	76	Average
Transferring data to high level is very easy	4	80	Good
Data do not need regular follow up	2.6	52	Poor
Registers and forms always available	4	80	Good
Laboratory media, solutions and equipment always available	2.7	52	Poor
No shortage in supplying drugs happened	3.5	70	Average
Average	3.6	70	Average

^a^MDR: multidrug resistant.

### Acceptability

All the respondents indicated that they receive quarterly incentives for their work and that they are willing to continue participating in the MDR-TB surveillance system. Overall, 81% of the respondents were satisfied. Acceptability scored 82%, which indicates that the system has good acceptability (Table 5).

### Data Quality

By reviewing the regional Sana’a city DR-TB center’s registers and cases files, we found that 5% and 20% had missing data, respectively. However, comparison of data on case files and register did not show any discrepancies.

### Overall Performance of the Multidrug-Resistant Tuberculosis Surveillance System

The performance of the surveillance system is illustrated in Table 6.

**Table 5:** The acceptability of the multidrug-resistant tuberculosis surveillance system as assessed by 16 service providers in Yemen.

Statements	Mean score	Percentage score	Rank
You are willing to participate in MDR-TB^a^ surveillance	4.3	86	Good
You are satisfied with MDR^b^ surveillance	3.9	79	Average
Average	4.1	82	Good

^a^MDR-TB: multidrug-resistant tuberculosis.

^b^MDR: multidrug resistant.

**Table 6:** Performance of the multidrug-resistant tuberculosis surveillance system.

Attributes	Performance	
	Percentage score	Rank
Usefulness	87	Good
Flexibility	61	Average
Stability	55	Poor
Simplicity	72	Average
Acceptability	82	Good
Data quality	91	Good
Total	74	Average

## Discussion

MDR-TB is a growing concern for TB programs, especially in developing countries [13]. Therefore, evaluation of MDR-TB surveillance is crucial. Our findings demonstrated good performance regarding the usefulness, especially for estimating the need for drugs to ensure case enrollment. It is also useful to ensure proper diagnosis and treatment for MDR-TB cases, which is one of the main goals for NTCP.

Based on the findings of this study, the flexibility of the system is average, as it can accommodate changes in case definitions and any updated guidelines. For example, case definition of rifampicin-resistant TB was added after GenXpert was introduced as a recommendation by the WHO [14]. Similar findings were reported from a previous evaluation in Pakistan that found that the MDR-TB surveillance can integrate with other systems [15].

Regarding the stability, we found that the MDR-TB surveillance system is unstable, mainly due to its sole dependence on donor support with a lack of governmental support. Furthermore, the frequent electricity cutoff in addition to the shortage of laboratory equipment at the regional centers negatively influenced the performance and made the system unstable, thereby affecting its sustainability. Therefore, constant power supply should be ensured for labs and appropriate laboratory equipment are essential.

The MDR-TB surveillance system seems like a simple system. Nevertheless, multiple registers and reports and the lack of a computerized system could make data collection more difficult. The acceptability of MDR-TB surveillance is good, which is reflected through the good data quality. The good acceptability might be a result of quarterly incentives for staff. However, cessation of such incentives will negatively impact acceptability and data quality. Previous similar evaluation in Pakistan found that both data quality and acceptability are good.

This study has some limitations. It did not shed light on the quantitative attributes and did not cover XDR-TB. Those limitations are attributed to the unavailability of all required lab tests for XDR-TB in addition to the specificity of MDR-TB that leads to inapplicability of some lab-related quantitative attributes.

In conclusion, the MDR-TB surveillance system provides useful data. However, dependence on donor funds affects stability and sustainability. MDR-TB centers or units should be expanded to cover all governorates. It is also recommended that constant power supply for labs is supplied and that there is a decrease in sole dependence on donor support through gradual replacement with governmental support.

## Abbreviations

DST: drug susceptibility testing
MDR: multidrug resistant
MDR-TB: multidrug-resistant tuberculosis
NTCP: National Tuberculosis Control Program
WHO: World Health Organization
XDR-TB: extensively drug-resistant tuberculosis
